# Supplementary material for: Mapping the Proteome of the Synaptic Cleft through Proximity Labeling Reveals New Cleft Proteins
Source: Proteomes. 2018 Nov 28;6(4):48. doi: 10.3390/proteomes6040048 (PMC6313906; doi:10.3390/proteomes6040048)
Supplement: Supplementary file 1 [file proteomes-06-00048-s001.zip › proteomes-381145-SI.pdf]

Supplementary Materials

Table S1. Synaptic cleft candidates identified by proximity labeling with SynCAM 1-HRP.

Protein	Gene	Repl.	Molecular Class	Enrichment	Specific
D166 antigen	Alcam	4	Adhesion molecule	2.65	3
Coxsackievirus and adenovirus receptor homolog	Cxadr	4	Cell surface receptor	2.95	1
Cadherin-2/N-cadherin	Cdh2	4	Adhesion molecule	2.10	1
Neurofascin	Nfasc	4	Adhesion molecule	1.84	1
Opioid-binding protein/cell adhesion molecule	Opcml	4	Adhesion molecule	1.68	1
Adhesion G protein-coupled receptor L3/Latrophilin 3	Adgrl3	4	Unclassified	1.50	1
Neural cell adhesion molecule L1	L1cam	4	Adhesion molecule	2.05	0
Neural cell adhesion molecule 1	Ncam1	4	Adhesion molecule	1.45	0
Contactin-1	Cntn1	4	Adhesion molecule	1.40	0
Neuronal growth regulator 1	Negr1	4	Unclassified	1.10	0
Neogenin (Fragment)	Neo1	3	Cell surface receptor	N.A.	3
Tyrosine-protein phosphatase non-receptor type substrate 1	Sirpa	3	Cell surface receptor	N.A.	3
Contactin-5	Cntn5	3	Unclassified	N.A.	3
Adhesion G protein-coupled receptor L1	Adgrl1	3	G protein coupled receptor	2.89	2
SynCAM 3/Cell adhesion molecule 3	Cadm3	3	Immunoglobulin	2.79	2
Voltage-dependent calcium channel subunit alpha-2/delta-1	Cacna2d1	3	Voltage gated channel	1.82	2
Neurexin-3	Nrxn3	3	Adhesion molecule	1.00	2
Cadherin-4 (Fragment)	Cdh4	3	Adhesion molecule	2.26	1
Neuroligin-2	Nlgn2	3	Integral membrane protein: Cell adhesion molecule activity	1.62	1
Noelin	Olfm1	3	Unclassified	1.04	1
Neuronal cell adhesion molecule	Nrcam	3	Adhesion molecule	2.23	0
Receptor-type tyrosine-protein phosphatase zeta	Ptprz1	3	Receptor tyrosine phosphatase	1.21	0
Neurexin-1	Nrxn1	3	Cell surface receptor	0.84	0
Leucine-rich repeat-containing protein 4B	Lrrc4b	2	Unclassified	N.A.	2
Probable G-protein coupled receptor 158	Gpr158	2	G protein coupled receptor	N.A.	2
MAM domain-containing glycosylphosphatidylinositol anchor protein 2	Mdga2	2	Unclassified	N.A.	2
Gamma-enolase	Eno2	2	Enzyme: Hydratase	N.A.	2
Dipeptidyl aminopeptidase-like protein 6	Dpp6	2	Membrane transport protein	N.A.	2
Contactin-associated protein 1	Cntnap1	2	Cell surface receptor	3.34	1

Neuroligin-3	Nlgn3	2	Integral membrane protein: Cell adhesion molecule activity	1.86	1
Tenascin-R	Tnr	2	Extracellular matrix protein	2.39	1
OX-2 membrane glycoprotein	Cd200	2	Unclassified	0.44	1
Receptor-type tyrosine-protein phosphatase S	Ptprs	2	Receptor tyrosine phosphatase	2.96	1
Leucine-rich glioma-inactivated protein 1	Lgi1	2	Unclassified	1.49	1
Gamma-aminobutyric acid receptor subunit gamma-2	Gabrg2	2	Extracellular ligand gated channel	0.98	1
Reticulon-4 receptor-like 2	Rtn4rl2	2	Cell surface receptor	2.15	0
Sodium/potassium-transporting ATPase subunit beta-1	Atp1b1	2	ATPase	0.81	0
Neuroplastin	Nptn	2	Immunoglobulin	1.81	0
Hyaluronan and proteoglycan link protein 1	Hapln1	2	Extracellular matrix protein	0.86	0
Excitatory amino acid transporter 2	Slc1a2	1	Membrane transport protein	0.00	2
Ciliary neurotrophic factor receptor subunit alpha	Cntfr	1	Cell surface receptor	N.A.	1
SynCAM 2/Cell adhesion molecule 2	Cadm2	1	Adhesion molecule	N.A.	1
BMP/retinoic acid-inducible neural-specific protein 2	Brinp2	1	Unclassified	N.A.	1
Annexin A1	Anxa1	1	Calcium binding protein	N.A.	1
Neurexin-2	Nrxn2	1	Cell surface receptor	N.A.	1
Sodium-driven chloride bicarbonate exchanger	Slc4a10	1	Membrane transport protein	N.A.	1
Neuronal pentraxin receptor	Nptxr	1	Cell surface receptor	N.A.	1
Plasminogen receptor (KT)	Plgrkt	1	Unclassified	N.A.	1
Solute carrier family 12 member 5	Slc12a5	1	Membrane transport protein	N.A.	1
Glutamate receptor ionotropic, NMDA 1	Grin1	1	Ion channel	N.A.	1
Solute carrier family 2, facilitated glucose transporter member 3	Slc2a3	1	Membrane transport protein	N.A.	1
DOMON domain-containing protein FRRS1L	Frrs1l	1	Integral membrane protein	N.A.	1
Vesicular inhibitory amino acid transporter	Slc32a1	1	Membrane transport protein	N.A.	1
Neurotrimin	Ntm	1	Adhesion molecule	3.76	0
V-type proton ATPase subunit C 1	Atp6v1c1	1	ATPase	2.01	0
Amyloid-like protein 2	Aplp2	1	Integral membrane protein	1.41	0
Cluster of Gamma-aminobutyric acid receptor subunit beta-3	Gabrb3	1	Extracellular ligand gated channel	1.23	0
Profilin-1	Pfn1	1	Cytoskeletal associated protein	3.50	0
Serum albumin	Alb	1	Transport/cargo protein	2.51	0

Coiled-coil domain-containing protein 80	Ccdc80	1	Unclassified	0.81	0
Leukocyte surface antigen CD47	Cd47	1	Unclassified	1.69	0
Prohibitin	Phb	1	Adapter molecule	0.53	0
Excitatory amino acid transporter 1	Slc1a3	1	Transport/cargo protein	0.45	0
Cluster of Glutamate receptor 2	Gria2	1	Extracellular ligand gated channel	1.14	0
Sodium- and chloride-dependent GABA transporter 1	Slc6a1	1	Membrane transport protein	0.51	0

Protein and gene names of synaptic cleft candidates are indicated, together with their Molecular Class according to the Human Protein Reference Database (HPRD; hdprg.org) and average synaptic cleft enrichment over plasma membrane where it passed Filter 1 and 2. Indicated is also how many biological replicates a protein was enriched (Replicates) and how often it was only detected in condition 5 (“Specific”).

Table S2. Comparison of synaptic cleft candidates identified by proximity labeling with SynCAM 1-HRP with excitatory synaptic cleft parts list of [29] Proteins shown were solely enriched in this study.

Protein	Gene	Repl.	Molecular Class
CD166 antigen	Alcam	4	Adhesion molecule
Neurofascin	Nfasc	4	Adhesion molecule
Adhesion G protein-coupled receptor L3/Latrophilin-3	Adgrl3	4	N.A.
Neogenin (Fragment)	Neo1	3	Cell surface receptor
Tyrosine-protein phosphatase non-receptor type substrate 1	Sirpa	3	Cell surface receptor
Adhesion G protein-coupled receptor L1	Adgrl1	3	G protein coupled receptor
Receptor-type tyrosine-protein phosphatase zeta	Ptprz1	3	Receptor tyrosine phosphatase
Gamma-enolase	Eno2	2	Enzyme: Hydratase
Dipeptidyl aminopeptidase-like protein 6	Dpp6	2	Membrane transport protein
Tenascin-R	Tnr	2	Extracellular matrix protein
Leucine-rich glioma-inactivated protein 1	Lgi1	2	Unclassified
Neuroplastin	Nptn	2	Immunoglobulin
Excitatory amino acid transporter 2	Slc1a2	1	Membrane transport protein
Ciliary neurotrophic factor receptor subunit alpha	Cntfr	1	Cell surface receptor
Cell adhesion molecule 2	Cadm2	1	Adhesion molecule
Annexin A1	Anxa1	1	Calcium binding protein
Sodium-driven chloride bicarbonate exchanger	Slc4a10	1	Membrane transport protein
Plasminogen receptor (KT)	Plgrkt	1	N.A.
Solute carrier family 12 member 5	Slc12a5	1	Membrane transport protein
Solute carrier family 2, facilitated glucose transporter member 3	Slc2a3	1	Membrane transport protein
DOMON domain-containing protein FRRS1L	Frrs1l	1	Integral membrane protein
Vesicular inhibitory amino acid transporter	Slc32a1	1	Membrane transport protein
V-type proton ATPase subunit C 1	Atp6v1c1	1	ATPase
Amyloid-like protein 2	Aplp2	1	Integral membrane protein
Profilin-1	Pfn1	1	Cytoskeletal associated protein
Serum albumin	Alb	1	Transport/cargo protein
Coiled-coil domain-containing protein 80	Ccdc80	1	Unclassified
Leukocyte surface antigen CD47	Cd47	1	Unclassified
Prohibitin	Phb	1	Adapter molecule
Excitatory amino acid transporter 1	Slc1a3	1	Transport/cargo protein

Protein and gene names of synaptic cleft candidates are indicated, together with their Molecular Class (HPRD). Indicated is also how many biological replicates a protein was enriched (Replicates).

Table S3. Candidate excitatory synaptic cleft orphans.

Protein	Gene	Repl.	Molecular Class
CD166 antigen	Alcam	4	Adhesion molecule
Adhesion G protein-coupled receptor L3/Latrophilin-3	Adgrl3	4	N.A.
Neogenin (Fragment)	Neo1	3	Cell surface receptor
Gamma-enolase	Eno2	2	Enzyme: Hydratase
Dipeptidyl aminopeptidase-like protein 6	Dpp6	2	Membrane transport protein
Tenascin-R	Tnr	2	Extracellular matrix protein
Leucine-rich glioma-inactivated protein 1	Lgi1	2	Unclassified
Ciliary neurotrophic factor receptor subunit alpha	Cntfr	1	Cell surface receptor
Annexin A1	Anxa1	1	Calcium binding protein
Sodium-driven chloride bicarbonate exchanger	Slc4a10	1	Membrane transport protein
Plasminogen receptor (KT)	Plgrkt	1	N.A.
Solute carrier family 12 member 5	Slc12a5	1	Membrane transport protein
V-type proton ATPase subunit C 1	Atp6v1c1	1	ATPase
Amyloid-like protein 2	Aplp2	1	Integral membrane protein
Profilin-1	Pfn1	1	Cytoskeletal associated protein
Coiled-coil domain-containing protein 80	Ccdc80	1	Unclassified
Prohibitin	Phb	1	Adapter molecule

Protein and gene names of synaptic cleft candidates are indicated, together with their Molecular Class (HPRD). Indicated is also how many biological replicates a protein was enriched (Replicates).

Table S4. GO terms used in post hoc in-silico filtering/data analysis.

Cellular Component
Golgi apparatus
cell surface
cytoplasm
cytoskeleton
endoplasmic reticulum
endosome
extracellular matrix
extracellular membrane-bounded organelle
extracellular region
extrinsic component of presynaptic membrane
extrinsic component of spine apparatus membrane
intracellular organelle
intracellular part
intrinsic component of presynaptic membrane
intrinsic component of spine apparatus membrane
mitochondrion
nucleus
organelle membrane
organelle part
plasma membrane
postsynapse
postsynaptic density, intracellular component
postsynaptic membrane
postsynaptic specialization
postsynaptic specialization membrane
postsynaptic specialization, intracellular component
presynapse
presynaptic active zone
presynaptic active zone membrane
presynaptic cytosol
presynaptic membrane
ribosome
spine apparatus
spine apparatus membrane
synapse
synapse part

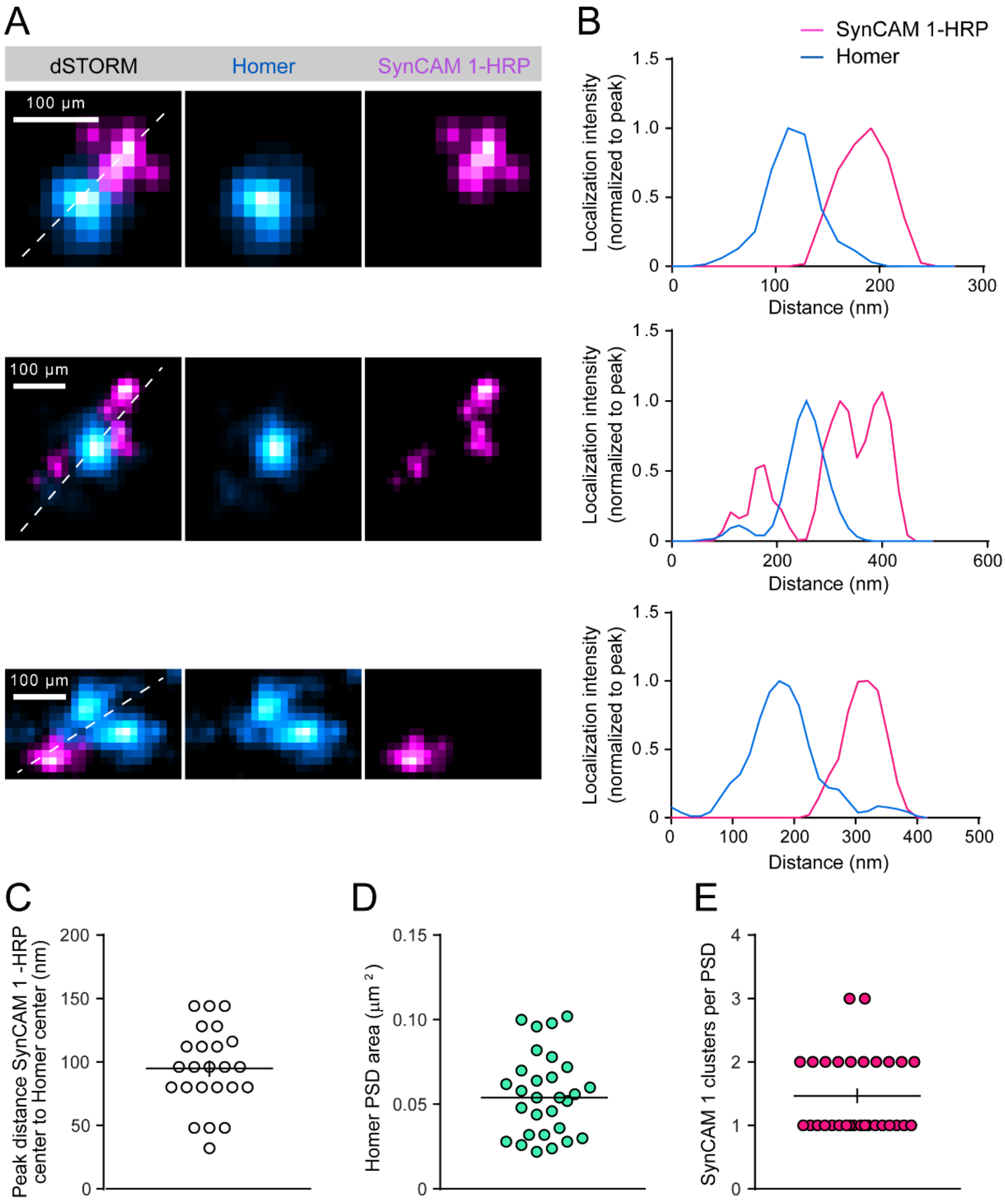

Figure S1. Characterization of super-resolved SynCAM 1-HRP and post-synaptic Homer. (**A**) Three examples of two-color dSTORM reconstruction of synapses from 21 days in vitro (div) rat hippocampal neurons surface-labeled by immunostaining with anti-FLAG antibodies against exogenous SynCAM 1-HRP containing the FLAG epitope (magenta) and the endogenous excitatory postsynaptic marker Homer (cyan). Diagonal line, line scan used for (B). (**B**) Protein localization distribution perpendicular to the trans-synaptic axis. Densities were determined by dSTORM and normalized to the peak of each channel and measured over the distance shown in A. (**C**) Inter-cluster distance analysis between center of SynCAM 1-HRP and post-synaptic Homer clusters. (**D**) Post-synaptic density (PSD) area as measured by size of Homer clusters. (**E**) Number of SynCAM 1-HRP clusters per PSD (Homer).
